# *In vitro* mouse spermatogenesis with an organ culture method in chemically defined medium

**DOI:** 10.1371/journal.pone.0192884

**Published:** 2018-02-12

**Authors:** Hiroyuki Sanjo, Mitsuru Komeya, Takuya Sato, Takeru Abe, Kumiko Katagiri, Hiroyuki Yamanaka, Yoko Ino, Noriaki Arakawa, Hisashi Hirano, Tatsuma Yao, Yuta Asayama, Akio Matsuhisa, Masahiro Yao, Takehiko Ogawa

**Affiliations:** 1 Department of Urology, Yokohama City University Graduate School of Medicine, Yokohama, Kanagawa, Japan; 2 Laboratory of Biopharmaceutical and Regenerative Sciences, Institute of Molecular Medicine and Life Science, Yokohama City University Association of Medical Science, Yokohama, Kanagawa, Japan; 3 Advanced Medical Research Center, Yokohama City University, Yokohama, Japan; 4 Department of Medical Life Science, Graduate School of Medical Life Science, Yokohama City University, Yokohama, Kanagawa, Japan; 5 Research and Development Center, Fuso Pharmaceutical Industries, Ltd., Osaka, Japan; Nanjing Medical University, CHINA

## Abstract

We previously reported the successful induction and completion of mouse spermatogenesis by culturing neonatal testis tissues. The culture medium consisted of α-minimum essential medium (α-MEM), supplemented with Knockout serum replacement (KSR) or AlbuMAX, neither of which were defined chemically. In this study, we formulated a chemically defined medium (CDM) that can induce mouse spermatogenesis under organ culture conditions. It was found that bovine serum albumin (BSA) purified through three different procedures had different effects on spermatogenesis. We also confirmed that retinoic acid (RA) played crucial roles in the onset of spermatogonial differentiation and meiotic initiation. The added lipids exhibited weak promoting effects on spermatogenesis. Lastly, luteinizing hormone (LH), follicle stimulating hormone (FSH), triiodothyronine (T3), and testosterone (T) combined together promoted spermatogenesis until round spermatid production. The CDM, however, was not able to produce elongated spermatids. It was also unable to induce spermatogenesis from the very early neonatal period, before 2 days postpartum, leaving certain factors necessary for spermatogenic induction in mice unidentified. Nonetheless, the present study provided important basic information on testis organ culture and spermatogenesis *in vitro*.

## Introduction

Fertility preservation is a critical issue in management of young cancer patients. Although cryopreservation of semen is an established method for male patients, it cannot be applied when male pediatric patients with malignant diseases, such as leukemia, are prepubescent. Thus, *in vitro* spermatogenesis induction from immature germ cells, if possible, may solve this problem [1]. There are two methods reported for *in vitro* spermatogenesis: organ culture and three-dimensional (3D) cell culture [2].

Organ culture experiments for *in vitro* spermatogenesis originated early in the 20th century [3]. Later, in the 1960’s, it was applied extensively and culture media improved, revealing several important factors for spermatogenesis [4]. However, spermatogenesis *in vitro* remained unsuccessful. In 2011, we reported the successful induction of mouse spermatogenesis in cultured neonatal testis tissues, leading to the formation of fertile sperm [5, 6]. The culture technique we used applied the classical gas-liquid interphase principle established in 1959 [7]. For our method, testis tissue fragments of approximately 1 mm^3^ were placed on an agarose gel block half-soaked in medium in a well. For the medium, we initially used fetal bovine serum (FBS) to supplement the basal medium, which induced spermatogenesis barely beyond the meiotic phase, and up to round spermatid formation in some cases [8]. This situation was significantly improved when FBS was replaced with KSR, which supported the progression of spermatogenesis up to the production of sperm [5]. We then found that AlbuMAX, a product of bovine serum albumin purified through a chromatography column, was equivalent with KSR in inducing and promoting spermatogenesis [5]. Both KSR and AlbuMAX are capable of inducing complete spermatogenesis in mouse testis tissues during the neonatal or fetal period [9]. Other research groups also reported that spermatogenesis was promoted in testis organ culture using medium containing either KSR or AlbuMAX in mice [10, 11, 12, 13, 14]. According to the manufacturing company, KSR includes 83 mg/mL of AlbuMAX [15]. The effector in KSR, therefore, may be AlbuMAX. Although AlbuMAX is a purified albumin product, it is not complete and contains several types of lipids [16, 17]. It may also be contaminated with serum-derived substances, including proteins, peptides, minerals, vitamins, and steroids. As addition of AlbuMAX to α-MEM induced full spermatogenesis, AlbuMAX contains all factors necessary for spermatogenesis. These factors, however, remain to be identified.

Spermatogenesis can be induced in the present organ culture system, but its efficiency is not comparable to that in the testis *in vivo*. For example, it was reported that the testis organ culture method, even with KSR or AlbuMAX, was unable to be used for *in vitro* screening of spermatogenic toxicity [11]. In addition, it is not effective in animal species other than mice, although it was marginally effective in inducing rat spermatogenesis [18]. Thus, the culture conditions, particularly the culture medium, have room for improvement and must be optimized for each species. The culture media commercially available at present have a long history of development, and are still being improved and new media are being created [19]. In order to improve the medium for spermatogenesis, we need to determine both non-specific-supportive and specific-promotive humoral factors. Chemically defined media containing such necessary factors offer an ideal platform for further improvements and adjustment for each species. The chemically defined medium may also help to elucidate the molecular mechanisms of spermatogenesis.

In this study, we aimed to produce a chemically defined medium, starting from the basal medium of α-MEM, through addition of defined ingredients. Although the formulated medium containing defined materials induced mouse spermatogenesis up to round spermatids, further improvements are necessary.

## Materials and methods

### Mice and treatments

*Acr-Gfp* transgenic mice (C57BL/6 strain) were provided by RIKEN BRC through the National Bio-Resource Project of MEXT, Japan. Female ICR, C57BL/6 (CLEA Japan), or ICRxC57BL/6 F1 mice were used for breeding, resulting in *Acr-Gfp* mice whose strain background is a mixture of ICR and C57BL/6. Male mice, *Acr-Gfp* (+/+) or (+/-), were used for the culture experiments. Most studies were performed using mice at 4.5 to 6.5 days post-partum (dpp) unless otherwise stated in the text. Spermatogenic cells in *Ac*r-*Gfp* mice express GFP from the mid-pachytene stage and onward. GFP accumulates and concentrates in the acrosome of spermatids. In the step 2–3 phase (early round spermatids), GFP appears as dot-like structures, then changes form into a crescent-like shape in the step 7–8 phase (late round spermatids). The crescent is distorted during spermatid elongation (step 10–11) (S1 Fig) [20]. All animal experiments conformed to the Guide for the Care and Use of Laboratory Animals, and were approved by the Institutional Committee of Laboratory Animal Experimentation (Animal Research Center of Yokohama City University, Yokohama, Japan).

### Culture method

Four BSA products were used: AlbuMAX® I (11020021, ThermoFisher Scientific), chromatographically-purified BSA (A2058, Sigma), Heat-shock fractionated BSA (A4919, Sigma), and Ethanol-fractionated BSA (A9418, Sigma). To make CDM, BSA was dissolved in double-distilled water (DDW) to yield a concentration of 80 mg/mL. Free fatty acids (FFAs), including Palmitic acid (P0500, Sigma), Oleic acid (O1008, Sigma), Linoleic acid (L1012, Sigma), and Linolenic acid (L2376, Sigma), were dissolved in ethanol at a 1,000x concentration as stock solution. Another FFA, Stearic acid (S4751, Sigma), was dissolved in ethanol at a 250x concentration as a stock solution. One-thousandth of each stock solution and 1/250 of Stearic acid stock solution were added to the BSA-dissolved water at final concentrations of 600, 600, 100, 50, and 600 μM, respectively. These concentrations were determined based on the concentration in the AlbuMAX medium (S1 Table). The solution was stirred for at least 4 hours at room temperature for complete dissolution. Then, the same volume of double-concentrated α-MEM, warmed to 35°C, was mixed with the BSA-FFA solution. Cholesterol (C3045, Sigma), Phosphatidylcholine (P3556, Sigma), and Sphingomyelin (S0756, Sigma) were also dissolved in ethanol at a 1,000x concentration as a stock solution, and added to the medium to achieve final concentrations of 3.2, 20.0, and 3.5 μg/mL respectively, according to a previous report [16]. Retinoic acid (R2625, Sigma. 10 mM stock sol. in DMSO), Retinol (R7632, Sigma. 10 mM stock sol. in DMSO), Testosterone (20808341, Wako. 10 mM stock sol. in DDW), Triiodothyronine (T6397, Sigma. 2.0 μg/mL stock sol. in DDW), LH (L5259, Sigma. 50 μg/mL stock sol. in DDW), and FSH (F4021, Sigma. 50 μg/mL stock sol. in DDW) were added to medium as indicated in the text to final concentrations of 1 μM, 1 μM, 1 μM, 2.0 ng/mL, 1.0 μg/mL, and 1.0 μg/mL, respectively. Finally, NaHCO_3_ was added to achieve a final concentration of 1.82 g/L (0.0182 g for 10 ml of medium), and 100x Antibiotic- Antimycotic at a final concentration of 100 IU/ml for penicillin, 100 μg/mL for streptomycin, and 250 ng/ml for amphotericin (15240062, ThermoFisher) was added to the medium. The media were stored at 4°C.

To make the agarose gel block for organ culturing, agarose powder was dissolved in DDW at 1.5% (w/v) and autoclaved. During the cooling, 33 mL of agarose solution was poured into 10-cm dishes to form a 5-mm thick gel. The gel was cut into approximately 10-mm-square pieces and these were used as stands for testis tissue placement. Gels were submerged in the culture medium for more than 6 hours before use in 12-well microplates. Testes were transferred to the surface of agarose gel that was half-soaked in 0.5 ml of medium in each well. Each gel stand was loaded with 2 to 4 tissues. The medium was changed once a week. The culture incubator was supplied with 5% carbon dioxide in air and maintained at 34°C. The protocol was reported previously in detail [21, 22].

### Hormone measurement

Luteinizing hormone and Follicle stimulating hormone were measured by chemiluminescence immunoassay (CLIA). Testosterone and Triiodothyronine were measured by electro-chemiluminescence immunoassay (ECLIA). These measurements were performed at SRL, Inc. (Tokyo). Retinoic acid was measured by high performance liquid chromatography (HPLC), performed at LSI medience corporation. (Tokyo)

### Lipids measurement

Free fatty acids included in AlbuMAX were measured by Gas chromatography–Mass spectrometry (GC-MS). Cholesterol was measured by ultraviolet absorption spectrophotometry (UV method). Phosphatidylcholine was measured by enzymatic method. These measurements were performed at SRL, Inc. (Tokyo).

### Observation

Cultured tissues were observed at least once a week under a stereomicroscope equipped with an excitation light for GFP (Leica M 205 FA; Leica, Germany). The observation of GFP expression along the tubules was designated as a sign of spermatogenesis, and it was classified into 6 grades, 0 ~ 5, based on the expression area: 0, ~20, ~40, ~60, ~80, and ~100%, respectively (S2 Fig). The central area, however, was omitted from the evaluation because this area in many cases lacked GFP expression due to spatial and nutrient flow restrictions. This GFP grading scale faithfully corresponded to spermatogenic progression, as confirmed in previous studies [5, 23].

To identify haploid cells with an acrosome cap structure, cultured tissues were observed with an inverted microscope applying the GFP-excitation light (Olympus IX 73; Olympus, Tokyo, Japan).

### Histological examination

Specimens were fixed with Bouin’s fixative and embedded in paraffin. One section showing the largest cut surface was made for each specimen, and stained with hematoxylin and eosin (H&E) or Periodic-Acid Schiff (PAS). For immunofluorescence staining, tissues were fixed with 4% paraformaldehyde in PBS overnight at 4°C. Cryoprotection was then performed with solutions of 10, 15, and 20% (w/v) sucrose in PBS for 1 hour each in succession. Tissues were cryo-embedded in OCT compound (Sakura Finetechnical) and cut into 7-μm-thick sections. The primary antibodies used were rat anti-Tra98 antibody (1:500, 2 μg/ml; Bioacademia), rabbit anti-Stra8 antibody (1:1,500, 667 ng/ml; Abcam), rabbit anti-Sycp1 antibody (1:200, 5 μg/ml; Novus Biologicals), and rabbit anti-γH2AX antibody (1:500, 100 ng/ml; Abcam). Tra98 is a pan-germ cell marker. Sra8 and γH2AX were used for detecting initial phase of meiosis. Sycp1 indicates pachytene spermatocytes. For the secondary antibodies, Alexa Fluor 555-conjugated goat anti-rat was used for the anti-Tra98 antibody, Alexa Fluor 488-conjugated goat anti-rabbit was used for the anti-Stra8 and anti-γH2AX antibodies, and Alexa Fluor 555-conjugated goat anti-rabbit was used for the anti-Sycp1 antibody. Nuclei were counterstained with Hoechst33342 dye.

### Statistical analysis

Non-parametric multiple comparison tests (Steel-Dwass) were performed to assess differences in the GFP expression grade. P < 0.05 was considered to indicate a significant difference. The rate of meiotic cell-containing tubules between two culture medium-groups (AlbuMAX and CDM) was examined with Mann–Whitney *U* test.

## Results

### Column-purified BSAs alone can induce spermatogenesis

**Fig 1:**
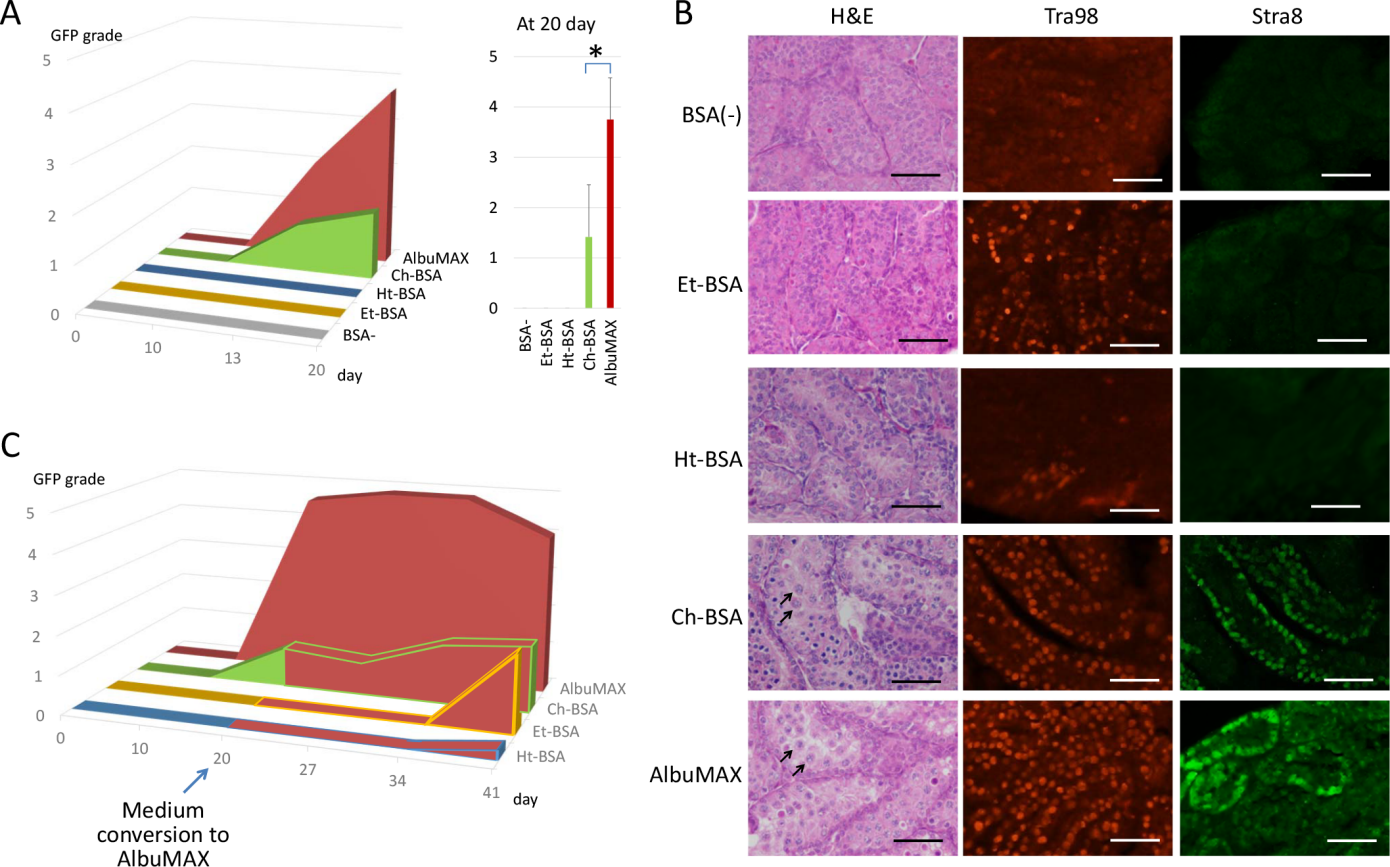
Spermatogenesis-inducing effects of BSAs purified by different methods. **A**) GFP expression grade time-course in mouse testis tissues cultured with 4 BSA products. Asterisk indicates P<0.01. **B**) Histological appearance of testis tissues cultured for 20 days with each BSA. Typical pachytene-stage spermatocytes are indicated with arrows. Immunohistochemistry was performed with a pan-germ cell marker (Tra98, Red) and meiosis marker (Stra8, Green). **C**) GFP expression grade time-course for 41 days (corresponding to 46 dpp). All media in each group were converted to media containing AlbuMAX on culture day 20.

In our culture system, AlbuMAX was critical for the induction and maintenance of *in vitro* spermatogenesis. However, whether the albumin molecule itself contributed remains unknown. Then, other BSA products were tested as to whether they had such effects on *in vitro* spermatogenesis. In general, there are three different purification procedures for albumin: ethanol fractionation, heat shock fractionation, and chromatographic concentration. Three BSA products corresponding to each procedure were selected, referred to as Et-BSA, Ht-BSA, and Ch-BSA, respectively. According to the purification procedures, the 3 BSAs have their own characteristics [24]. The ethanol fractionation method, also called Cohn’s method, is the most popular. This method includes processing at low temperatures (-5 ~ -3°C), pH adjustment (4.8 ~ 7.2), and ethanol addition (8 ~ 40%). By using ethanol at high concentrations, insoluble substances like lipids are mostly eliminated. The heat shock method is a modified version of the ethanol method that includes a heating process (68°C) with 9% ethanol at pH 6.5, along with caprylate as a protein stabilizer to denature and precipitate proteins other than albumin. The chromatographic method uses ion- exchange and gel filtration chromatography, and neither ethanol nor heat is applied. Thus, this method may be milder for proteins like albumin than the previous two methods, but it is prone to minor contamination from several substances, including lipids from the source serum. When the testis tissues of *Acr-Gfp* transgenic mice were cultured in medium containing each BSA individually, only AlbuMAX and Ch-BSA induced GFP expression (Fig 1A). Histological examination identified meiotic figures and expansion of the seminiferous tubules only in samples cultured with AlbuMAX or Ch-BSA. In contrast, samples cultured with Ht-BSA or Et-BSA or without BSA consisted of seminiferous tubules with a thinner diameter, no lumen, and no meiotic figures; they remained fully immature (Fig 1B). On immunohistological examination with the Stra8 antibody, differentiating spermatogonia and early-stage meiotic cells were identified as Stra8-positive cells in samples cultured in AlbuMAX or Ch-BSA on culture day 20. On the other hand, on staining for Tra98, which is a pan-germ cell marker, positive cells were identified in all samples. However, media containing AlbuMAX or Ch-BSA seemed to maintain more Tra98-positive cells than the other media (Fig 1B). In order to check if the Tra98-positive cells, likely undifferentiated spermatogonia, in tissue samples cultured with media containing Et-BSA or Ht-BSA were functional or not, the medium was changed to AlbuMAX-containing medium on day 20. Consequently, GFP expression was observed at day 41 in the Et-BSA and Ht-BSA samples, and in higher levels with Et-BSA, indicating that the spermatogonia maintained in the BSA-media were functional (Fig 1C). Based on these observations, we decided to use Et-BSA for the following experiments to formulate a chemically defined medium because the basic structure of the testis tissue and spermatogonia were maintained better with Et-BSA than with Ht-BSA. Although Ch-BSA demonstrated spermatogenesis-induction ability, it was omitted from further CDM construction experiments because it was not pure enough and was contaminated with serum-derived substances, similar with AlbuMAX.

### RA signaling promoted spermatogonial differentiation up to meiotic entry

**Fig 2:**
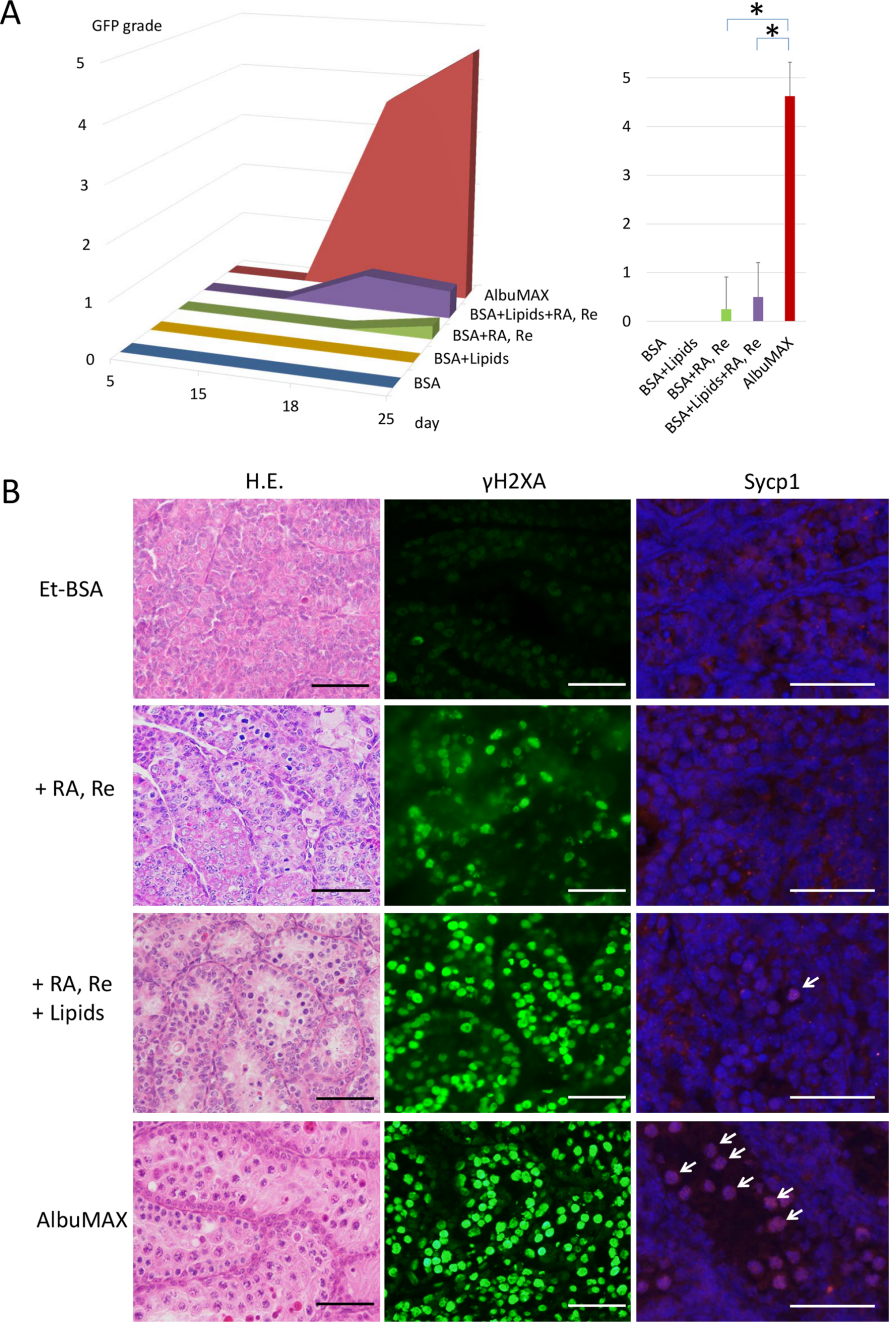
Effects of retinoic acid and lipids. **A)** Representative *Acr*-GFP expression grade time-course with the addition of RA and retinol (RA, Re), lipids, and both in the medium is shown in the graph. Asterisk indicates P<0.01. **B)** Histological examination with standard H.E. staining, and immunohistochemistry with antibodies for γH2AX and Sycp1 counterstained with Hoechst. Sycp1-positive cells, indicated by arrows, were pachytene-stage spermatocytes. Scale bar: 50 γm.

Retinoic acid (RA) is a critical factor for inducing the differentiation of undifferentiated spermatogonia and guiding them towards meiosis [25–31]. Thus, we added RA and retinol, the precursor of RA, to the medium containing Et-BSA. Although GFP expression was hardly observed (Fig 2A), histological examination revealed some meiotic figures, including leptotene-stage spermatocytes (Fig 2B). Immunohistological examination with the antibody for γH2AX identified positive signals consistent with leptotene-stage spermatocytes, whereas there were no Sycp1-positive cells, indicating that meiosis did not progress to the pachytene stage (Fig 2B). These immunohistological findings were consistent with the observation that *Acr-Gfp* expression was mostly negative, as it starts at the mid-pachytene stage. It was concluded therefore that RA and retinol supplementation to solely generate an RA signal can induce meiosis in undifferentiated spermatogonia. However, RA signaling in the basal medium and with Et-BSA was unable to advance meiosis beyond the meiotic initiation phase.

### Lipids support meiotic progression

AlbuMAX has been described as lipid-rich albumin. The lipid composition was also reported, which is critical for its effects on cultured cells to promote their proliferation [16]. We therefore speculated that spermatogenic induction may be attributed, at least in part, to lipids being present in AlbuMAX. Based on data in these two reports, we chose free fatty acids (FFA), cholesterol, phosphatidylcholine, and sphingomyelin as possible critical lipids. For FFA, we selected palmitic, stearic, oleic, linoleic, and linolenic acids. These lipids were mixed in the Et-BSA solution in addition to RA and retinol. Although the addition of these lipids alone to Et-BSA did not induce *Acr-*GFP expression, they improved the GFP expression level marginally when added with RA and retinol (Fig 2A). Histological examination confirmed the effects of the lipids, leading to a greater number of meiotic cells and seminiferous tubules containing meiotic cells. Immunohistology revealed an increase in leptotene-stage spermatocytes, γH2AX-positive cells, and the appearance of a small number of suspected pachytene-stage spermatocytes, Sycp1-positive cells, in the lipid-added culture samples (Fig 2B). These results suggest that lipids promote spermatogenesis under the organ culture conditions, especially in the meiotic entry phase.

### Hormones enhance spermatogenic progression

**Fig 3:**
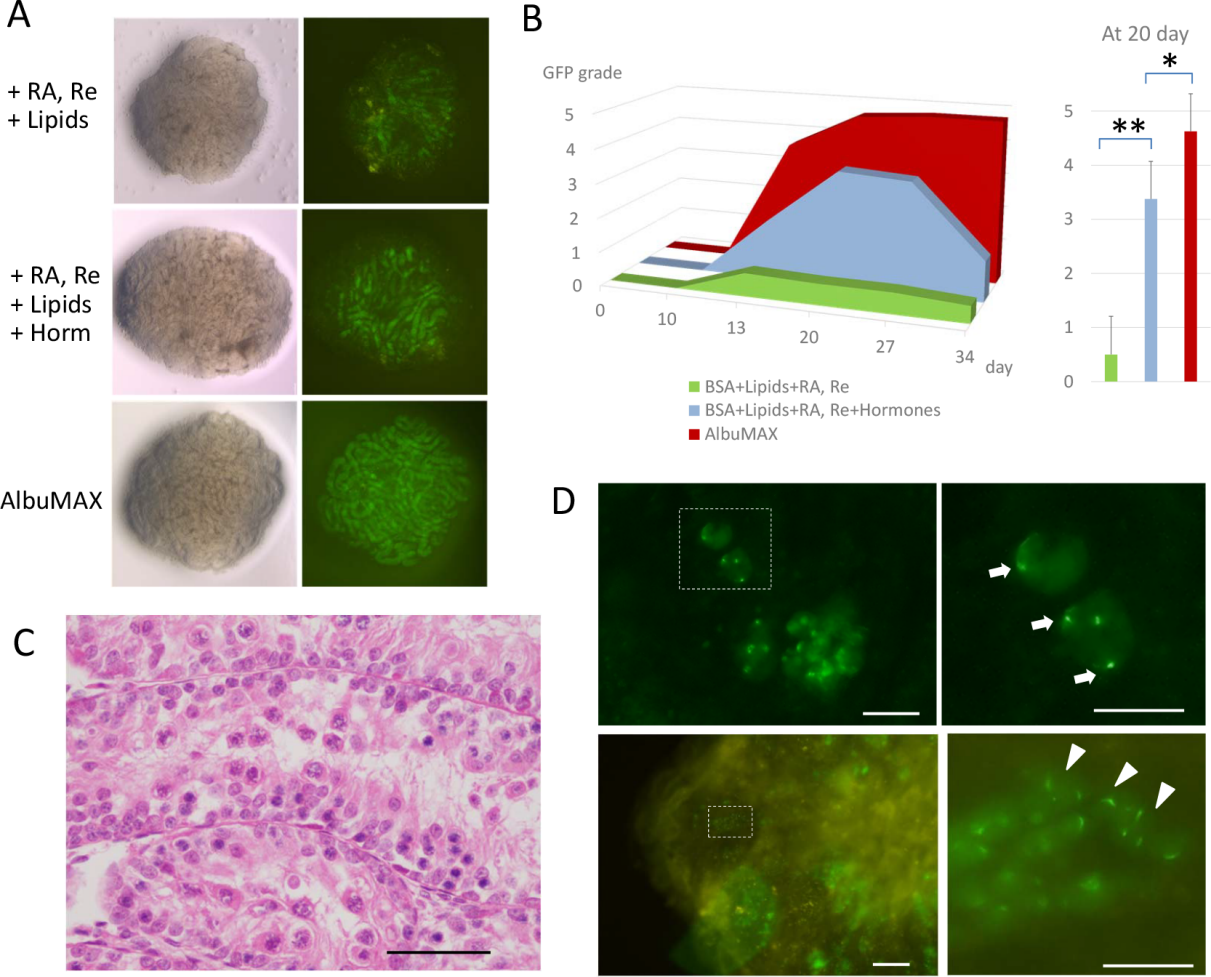
Effects of hormones. **A)** Stereomicrospic view of testis tissue obtained from a 5.5 dpp *Acr-Gfp* mouse cultured with BSA + RA, Re + lipids, BSA + RA, Re + lipids + hormones, and AlbuMAX for 15 days; a view of the sample lit from underneath (left) and with GFP-excitation light (right). **B)** The GFP grade time-courses of CDM containing BSA, RA, Re and lipids (green), hormones of T, LH, FSH, and T3, in addition to BSA, RA, Re, and lipids (light blue), and AlbuMAX (dark red). Single and double asterisks indicate P<0.05 and P<0.01, respectively. **C)** Histological appearance of cultured tissues with BSA including lipids, RA, and hormones on culture day 35. **D)** In dissociated tissues cultured with CDM containing the hormones, haploid cells were identified as exhibiting *Acr*-GFP condensation (arrow) and the formation of a cap-like structure (arrowhead). Dashed rectangles in the left panel are enlarged in the right panel. Scale bar: 50 γm.

There have been numerous reports on the hormonal control of spermatogenesis [32]. Testosterone, LH, and FSH have been the most extensively studied regarding their effects on spermatogenesis [33]. The effects of triiodothyronine (T3) on Sertoli cell maturation and spermatogenesis in the developmental period were also noted [34]. However, their effects under culture conditions have rarely been studied due to the lack of a culture system that can facilitate such evaluation. We considered these hormones to also have important roles in our culture system. Indeed, AlbuMAX contains these hormones, albeit at very low concentrations (S1 Table). In order to improve the spermatogenic progression *in vitro*, we added these hormones to the culture medium that already contained Et-BSA, RA, retinol, and lipids (S1 Table). The concentrations of testosterone, LH and T3 that we adopted were at physiological levels; they were at the intra-testicular level for LH and around serum level for the other two. Supplemented FSH was lower than the physiological level, but much higher than the concentration in the AlbuMAX-containing medium. The addition of these hormones greatly enhanced the GFP-expression (Fig 3A and 3B), even though still not comparable with the AlbuMAX-containing medium. Histologically, the diameter of the seminiferous tubules enlarged and pachytene spermatocytes became apparent (Fig 3C). Comparative histological examination between AlbuMAX medium and CDM on culture day 20 and 35 revealed that progression into meiotic phase was observed frequently in both groups, but this progression was much further and more wide-spreading in tissues cultured with AlbuMAX medium (S3 Fig). However, round spermatids were observed as GFP condensations or forming cap-like structures in cultured tissues, which correspond with early round spermatids (step 2–3) and late round spermatids (step 7–8), respectively (Fig 3D) [20]. From culture days 35 to 44, 86 tissues from 34 animals were dissociated and examined for haploid cells, revealing 51 (59.3%) and 15 (17.4%) tissues containing early and late round spermatids, respectively. However, no elongated spermatids were found (S2 Table).

### Importance of donor mouse age on culture results

Our final CDM formulation (S1 Table) induced mouse spermatogenesis up to round spermatid formation in the cultured testis tissues. We then tested the donor mouse age ranging from 1.5 dpp to 5.5 dpp. We noticed in this set of experiments that one of the most critical factors influencing the culture results was the age of mice used for testis tissue sampling. When younger mice, particularly at 2.5 dpp or younger, were used, the *Acr-*GFP expression area was significantly smaller (S4A Fig). The culture results also corresponded with the body weight of donor mice (S4B Fig). In particular, at an age of 1.5 dpp or weight of 2.0 g or lower, the testis tissues cultured in the chemically defined medium exhibited few signs of spermatogenesis. This markedly contrasted with the results from culturing with AlbuMAX. In addition, the levels of GFP expression declined faster with CDM than with AlbuMAX (Fig 3B). These unfavorable results with CDM indicate that some important factors are still missing.

## Discussion

In this study, we aimed to formulate a CDM without using AlbuMAX. First, we tested three different BSA products by adding them to the culture media, and assessed whether they had spermatogenesis-inducing effects like AlbuMAX. We found that chromatographically purified BSAs alone had such effects, but the two other BSAs purified through heat-shock or ethanol precipitation methods, respectively, had no effects. The marked difference between the chromatographically purified and two other methods was surprising, and suggested that it is not albumin, but rather the other substances contaminating the BSA product that are important. In particular, we considered that substances binding to albumin molecules may be critical. Indeed, both heat-shock and ethanol-precipitation methods degenerate albumin, which tends to disrupt binding. On the other hand, the chromatography method maintains the conformational structure of albumin throughout the process, and thus, substances will remain bound to albumin. Although we deduced that albumin itself does not exhibit spermatogenesis-inducing activity, its presence may be essential in the medium due to its binding with hydrophobic substances like lipids. We, therefore, concluded that BSA should also be included in the CDM formulation and chose to add Et-BSA to the formulation because it maintained spermatogonia more favorably than Ht-BSA.

It has been established that RA is the main effector inducing meiosis [31]. In particular, RA induces the transition of undifferentiated to differentiating spermatogonia, which is accompanied by the expression of genes such as Stra8 and c-kit [25–29]. In organ culture conditions, the effects of RA also improved the spermatogenic efficiency [12, 13]. Thus, RA, along with retinol, was added initially to the primitive medium composed of α-MEM and Et-BSA in order to assess the effects of RA on undifferentiated spermatogonia. On histological examination, we observed that the addition of RA and retinol induced the appearance of leptotene- or zygotene-stage spermatocytes, but never beyond. The *Acr-*GFP expression was weak in a few cases, corresponding with the histological findings. This suggested that the RA signal is sufficient for initiation of meiosis, but not for its completion. This is the first report to demonstrate the limited potential of RA to promote spermatogenic meiosis under organ culture conditions.

Previous reports emphasized that lipids are important ingredients in AlbuMAX that promote several biological activities [16, 17]. In general, lipids are essential for cells and organs as an energy source, material for bioactive products, and components of the cell membrane. As spermatogenic cells change their cell shape dynamically, particularly during spermiogenesis, the cell membrane composition also changes during this process. Indeed, docosahexaenoic acid (DHA) and arachidonic acid were reported to be essential for spermiogenesis [35]. In addition, there are increasing numbers of reports on the roles of lipids as signal mediators inducing specific activities in target cells. Phosphoinositides, such as phosphatidyl inositol 4, 5- bisphosphate (PIP2) and Inositol triphosphate (IP3), are prime examples that mediate the PI3 kinase pathway [36]. However, there have been few reports on their roles in spermatogenesis, particularly in its induction. In the present study, the addition of lipids to the CDM, which consisted of α-MEM, Et-BSA, RA, and retinol, led to stronger induction of meiosis, based on increased numbers of leptotene- and zygotene-stage spermatocytes. The mechanism by which lipids exert their effects on spermatogenic progression, however, remains unclear, and this warrants further precise examination.

Hormones like LH, FSH, and testosterone have been extensively studied with regard to spermatogenesis [32, 33]. Triiodothyronine (T3) has been recognized as important for the maturation of Sertoli cells [34]. Thus, we added these hormones to our CDM, which significantly improved the progression of spermatogenesis. Precisely, hormones influenced the meiotic spermatocytes that halted around the leptotene or zygotene stage, and promoted them through the pachytene stage up to meiotic division, resulting in round spermatid formation. The condensation of *Acr*-GFP into the acrosome structure followed by formation into a cap-like form is a reliable indication of round spermatid formation that was found in the tissue cultured in the CDM containing four hormones.

In summary, the CDM formula achieved in this study supported mouse spermatogenesis in cultured testis tissue. However, this was not observed using neonatal mice at younger ages of 0.5 to 2.5 dpp, or with body weights lighter than 2.0 g. This suggests that there are factors missing from the CDM that induce the very initial step of spermatogenesis, the differentiation of gonocytes or primitive spermatogonia. As stated above, RA has been reported to be a factor inducing the transition between undifferentiated and differentiating spermatogonia. However, the present study revealed that RA does not initiate spermatogenesis in immature neonatal mouse testes. Additionally, it is noteworthy that the spermatogenesis supported by the CDM did not last as long as that with AlbuMAX. The *Acr*-GFP expression showed rapid decline and extinguished, suggesting that the duration may only be a single cycle of spermatogenesis. This is consistent with the fact that CDM cannot induce spermatogenesis from the primitive spermatogonia of neonates. The CDM is not only incapable of inducing neonatal spermatogenesis, it also cannot induce the commitment to spermatogenesis in spermatogonial stem cells, making *in vitro* spermatogenesis only possible with committed spermatogonia.

In the present study, we developed a CDM and succeeded in producing haploid cells using an organ culture method. This is the first report, to our best knowledge, of successful *in vitro* spermatogenesis with a chemically defined medium. Although the present study leaves many questions unanswered, as stated above, the CDM formulation provides a basis for future studies and may help generate important information in this field of research.

## Supporting information

S1 Table Chemically-defined medium composition. Ingredients listed in the left–most column were added in α-MEM to produce the chemically-defined medium. The right-most column shows the concentration of each ingredient in α-MEM+AlbuMAX (40 mg/mL).
(TIF)

S2 Table The counts of haploid cells induced by AlbuMAX and CDM. Tissues were dissociated at 35 to 44 days of cultivation to identify haploid cell production based on GFP-positive acrosomal configuration under an inverted-microscope equipped with GFP-excitation light.
(TIF)

S1 Fig The characteristic acrosomal GFP condensation of *Acr*-Gfp transgenic mice. *Acr*-Gfp appears in the cytoplasm in meiotic cells. Then, it accumulates in the acrosome structure showing a condensation as a dot, which sequentially changes into a crescent-like structure. This GFP appearance corresponds to spermatid maturation.
(TIF)

S2 Fig GFP expression classification. GFP expression was classified into 5 grades, by percentage of GFP-expression area observed under a stereomicroscope.
(TIF)

S3 Fig Histological evaluation of spermatogenesis with two culture media, AlbuMAX and CDM. Representative histological appearance of testis tissues cultured with AlbuMAX medium and CDM on culture days 20 and 35. The rectangular area is enlarged in the left bottom corner. The rate of seminiferous tubules containing meiotic germ cells among all tubules in a section on day 20 and 35 are presented as a bar graph. Significant differences (P < 0.01) between these two medium groups were detected. Scale bar: 200 μm.
(TIF)

S4 Fig The limitations of chemically-defined medium. Testis tissue of mice aged 1.5 ~ 5.5 dpp was cultured with CDM. The data were also classified by body weight. CDM couldn’t induce spermatogenesis in low weight and low aged mice.
(TIF)
