# Supplementary figures and images for: In vitro mouse spermatogenesis with an organ culture method in chemically defined medium
Source: PLoS One. 2018 Feb 12;13(2):e0192884. doi: 10.1371/journal.pone.0192884 (PMC5809087; doi:10.1371/journal.pone.0192884)

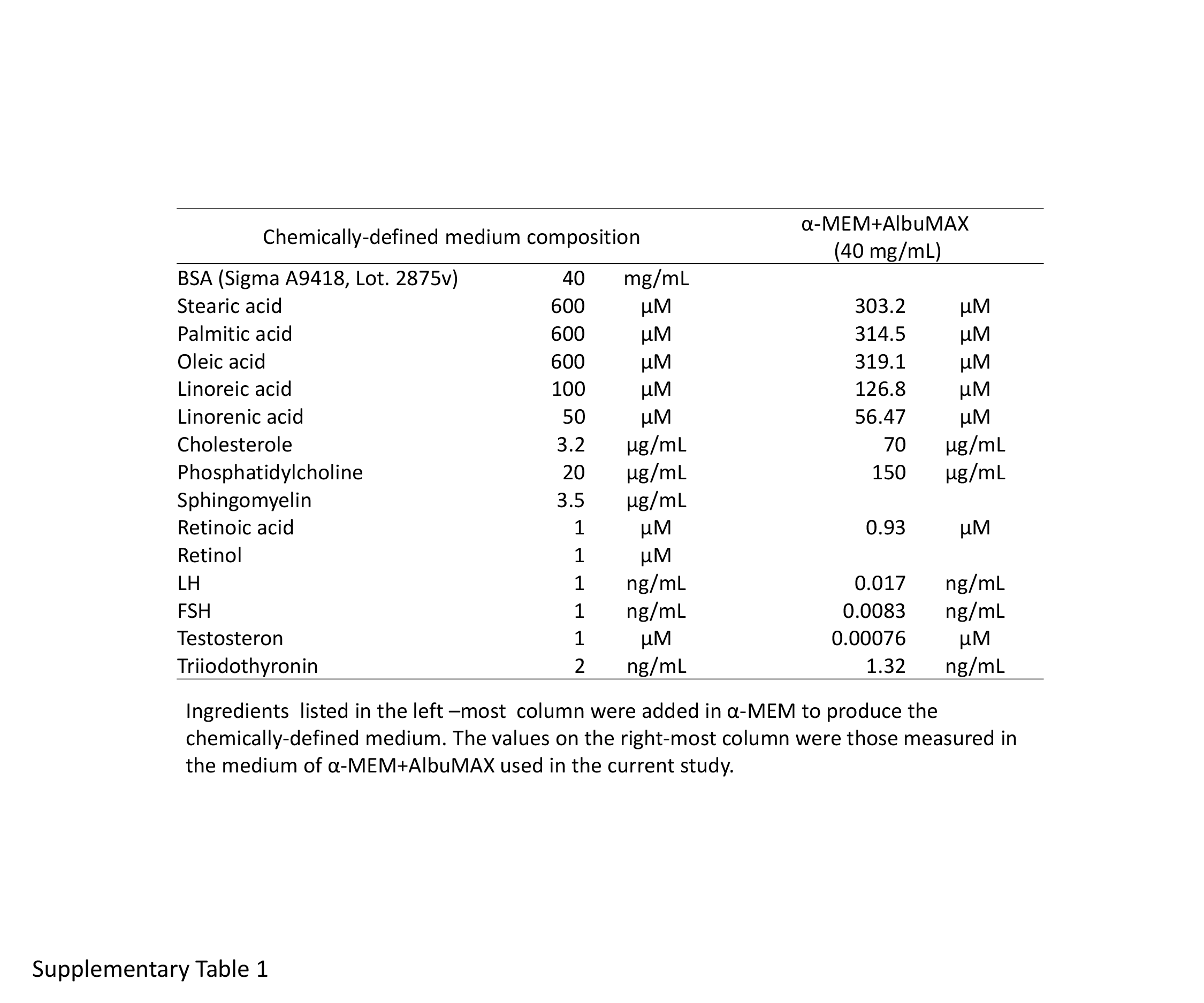

Supplement: S1 Table — Ingredients listed in the left–most column were added in α-MEM to produce the chemically-defined medium. The right-most column shows the concentration of each ingredient in α-MEM+AlbuMAX (40 mg/mL). (TIF) [file pone.0192884.s001.tif]

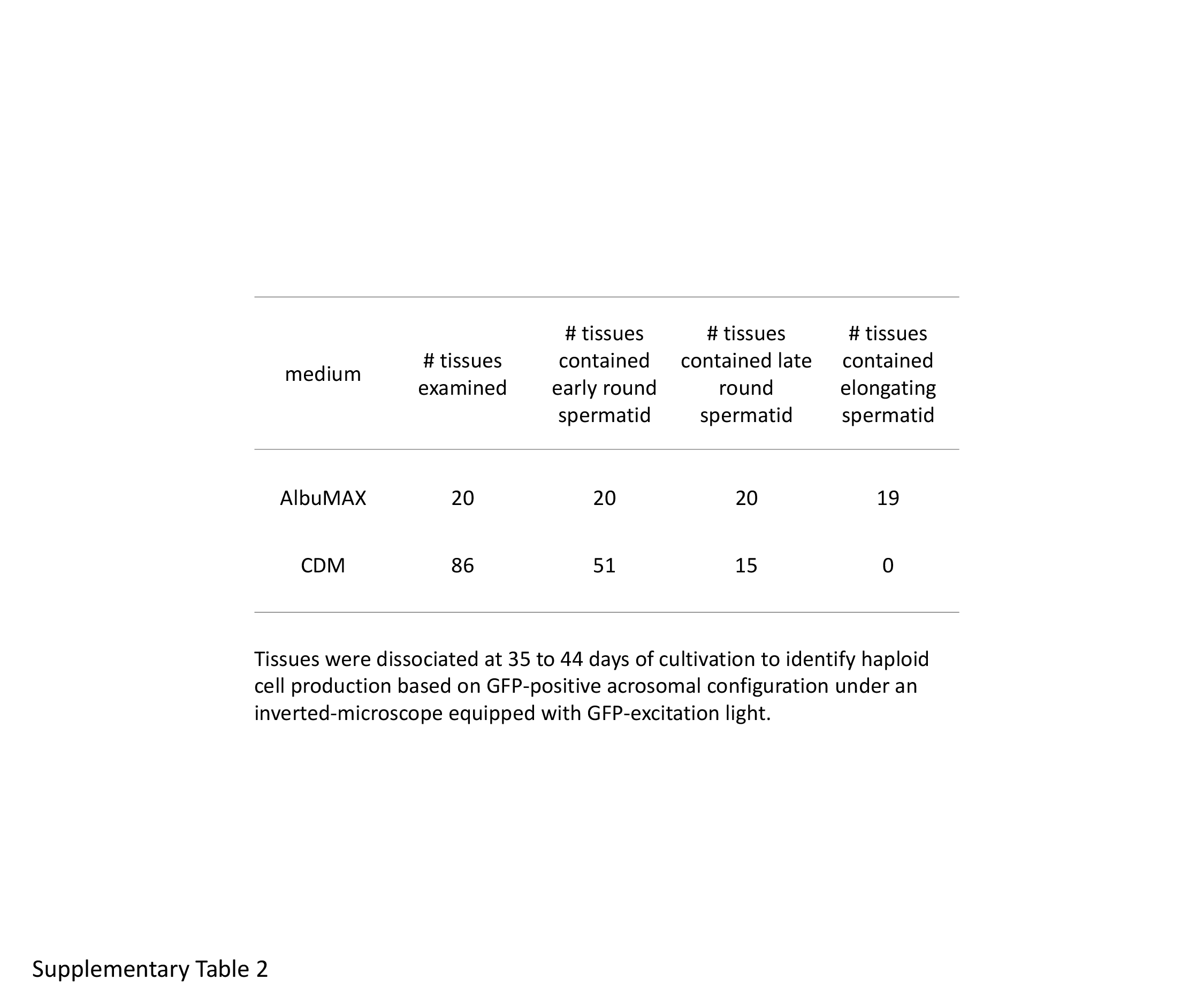

Supplement: S2 Table — Tissues were dissociated at 35 to 44 days of cultivation to identify haploid cell production based on GFP-positive acrosomal configuration under an inverted-microscope equipped with GFP-excitation light. (TIF) [file pone.0192884.s002.tif]

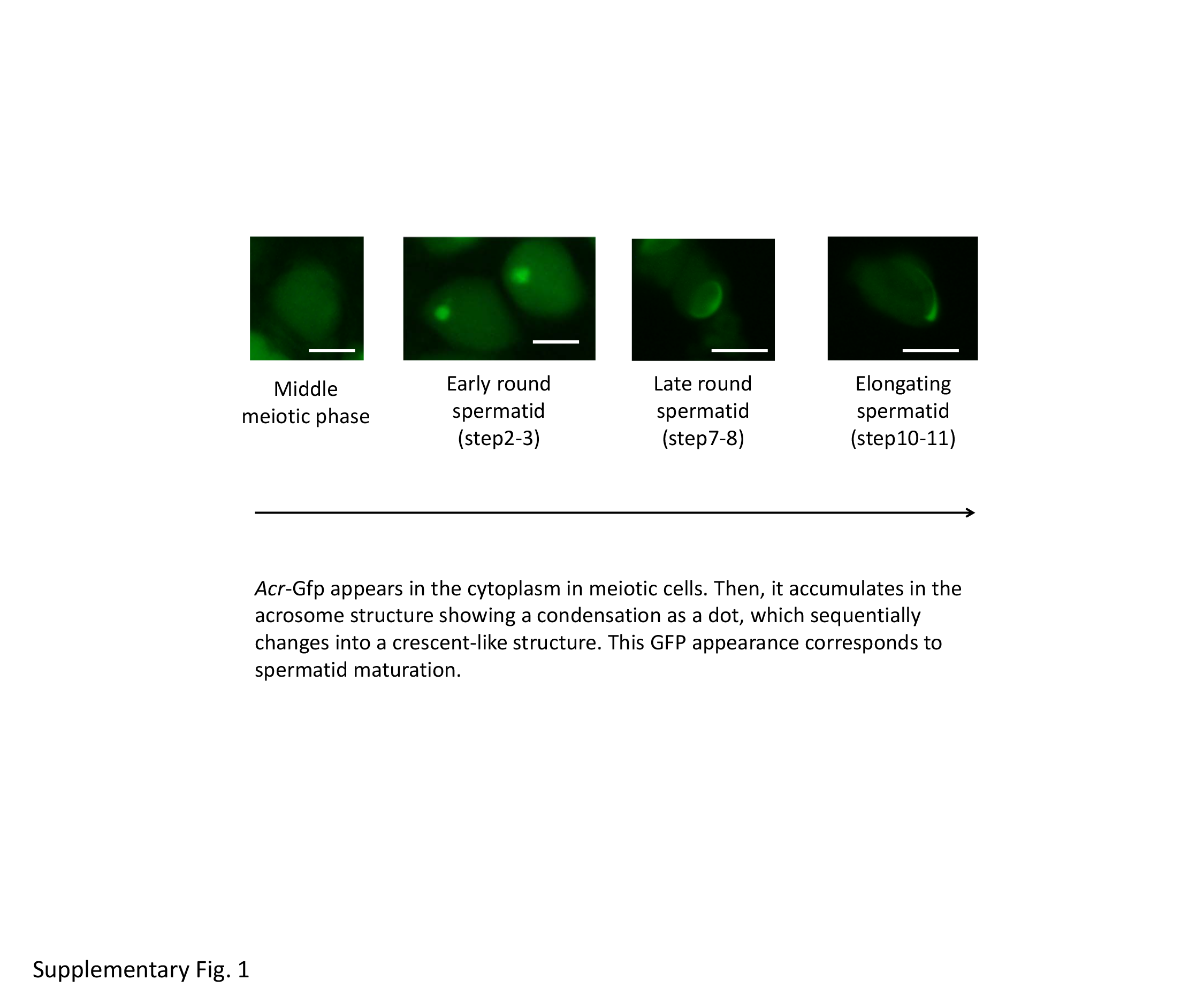

Supplement: S1 Fig — Acr-Gfp appears in the cytoplasm in meiotic cells. Then, it accumulates in the acrosome structure showing a condensation as a dot, which sequentially changes into a crescent-like structure. This GFP appearance corresponds to spermatid maturation. (TIF) [file pone.0192884.s003.tif]

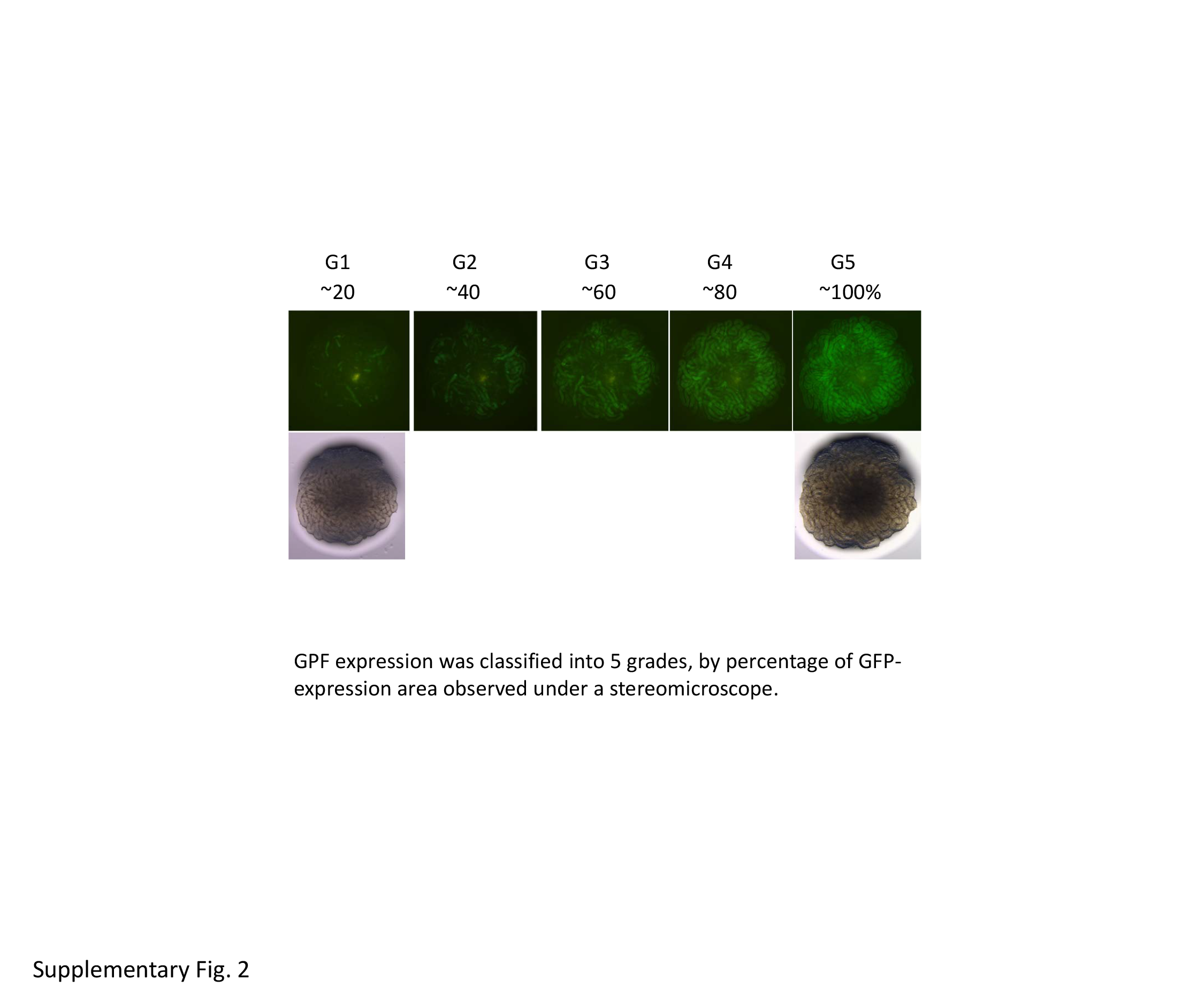

Supplement: S2 Fig — GFP expression was classified into 5 grades, by percentage of GFP-expression area observed under a stereomicroscope. (TIF) [file pone.0192884.s004.tif]

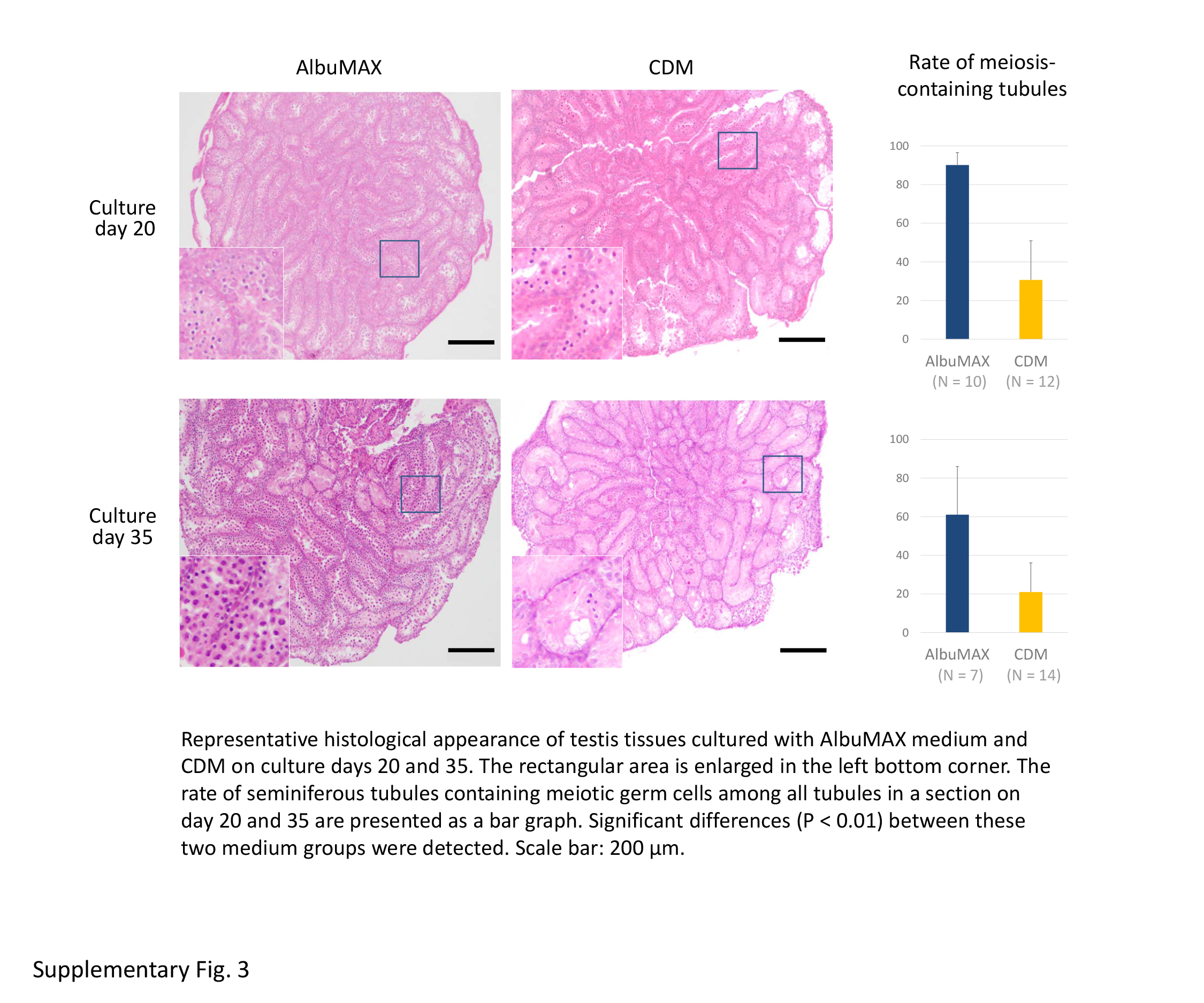

Supplement: S3 Fig — Representative histological appearance of testis tissues cultured with AlbuMAX medium and CDM on culture days 20 and 35. The rectangular area is enlarged in the left bottom corner. The rate of seminiferous tubules containing meiotic germ cells among all tubules in a section on day 20 and 35 are presented as a bar graph. Significant differences (P < 0.01) between these two medium groups were detected. Scale bar: 200 μm. (TIF) [file pone.0192884.s005.tif]

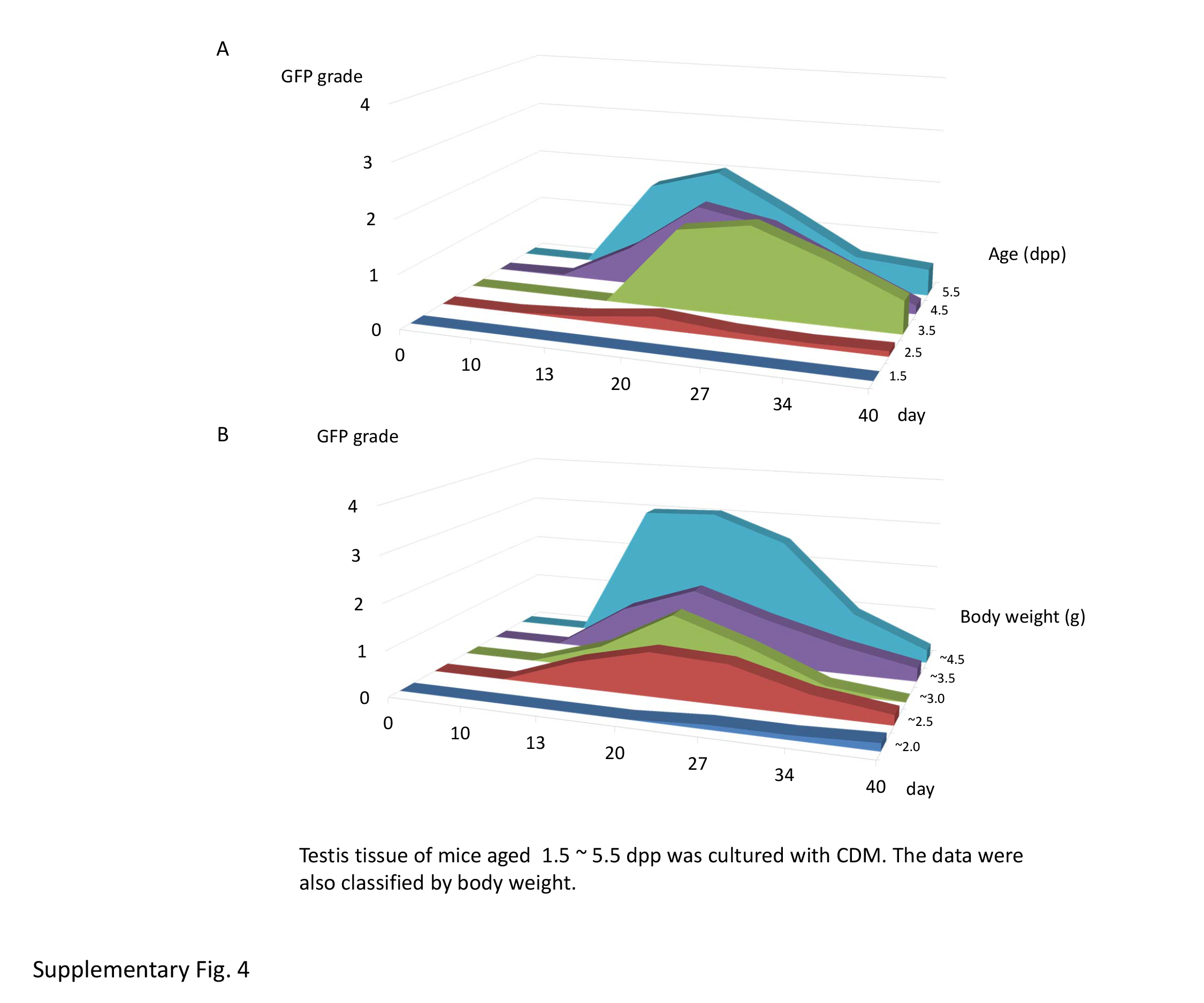

Supplement: S4 Fig — Testis tissue of mice aged 1.5 ~ 5.5 dpp was cultured with CDM. The data were also classified by body weight. CDM couldn’t induce spermatogenesis in low weight and low aged mice. (TIF) [file pone.0192884.s006.tif]
